# Whole-Exome Sequencing Analysis Identifies Risk Genes in Atlantoaxial Dislocation Patients with Sandwich Fusion

**DOI:** 10.1155/2024/5021689

**Published:** 2024-03-12

**Authors:** Guodong Gao, Yinglun Tian, Kan-Lin Hung, Dongwei Fan, Nanfang Xu, Shenglin Wang

**Affiliations:** Peking University Third Hospital Orthopaedics Department, Beijing, China

## Abstract

Sandwich fusion of Klippel-Feil syndrome (KFS), which is a rare congenital disorder involving the fusion of cervical vertebrae, poses significant challenges in the diagnosis and treatment of atlantoaxial dislocation (AAD). While the disorder's genetic basis is not well-understood, the rarity of the sandwich fusion makes it difficult to study. Whole-exome sequencing (WES) was conducted on 68 unrelated Chinese patients with sandwich fusion. The study compared their genetic data with a control group of 219 individuals without musculoskeletal disorders. Various analyses, including mutational burden assessments, were employed to identify potential pathogenic genes. The study identified significant genetic variations in patients with sandwich fusion, highlighting genes like *KMT5A*, *HYDIN*, and *PCDHB4* as potential contributors. Notably, severe cases exhibited oligogenic effects, with mutations in genes like *MEOX1* associated with the severity of spinal issues. These findings offer critical insights into the genetic basis of sandwich fusion and provide a foundation for future research and therapeutic development.

## 1. Introduction

Atlantoaxial dislocation (AAD) is a rare and potentially life-threatening anatomical disturbance of the craniovertebral junction, characterized by the loss of stability between the atlas (C1) and axis (C2) due to traumatic, inflammatory, idiopathic, or congenital abnormalities. This instability leads to dislocation of the normal joint, and if not promptly and appropriately treated, it may result in permanent central nervous system dysfunction, such as high quadriplegia [1]. One of the susceptible populations for AAD is the sandwich fusion anomaly, which refers to congenital spinal fusion affecting both C0-1 and C2-3 simultaneously, presenting a significantly increased risk of AAD and subsequent high cervical cord events [2, 3].

The sandwich anomaly represents a specific subtype of Klippel-Feil syndrome, a congenital disorder characterized primarily by segmental fusion of cervical vertebrae and diagnosed through radiographic imaging [4]. KFS has long been considered a rare disorder, with an estimated incidence of approximately 1 in 40,000 live births and a higher prevalence in female infants [5]. However, clinicians widely acknowledge the likelihood of underreporting in this data [6]. Clinical manifestations of KFS exhibit significant heterogeneity, ranging from isolated cervical spine fusion to associated anomalies in other organs or systems. Fusion sites may involve single or multiple fusions at various locations, including continuous long fusions [7]. The most common fusion sites are C2-3 and C5-6, with C2-3 fusion accounting for 74.1% of congenital cervical fusions [8]. The sandwich fusion, a distinctive subtype of KFS, concentrates compensatory stress between C1 and C2, making it highly prone to AAD, leading to spinal cord pathology and even concurrent cranial nerve disorders. Currently, there are no effective predictive tools or supportive therapies for sandwich fusion, making exploration of its pathogenic genetic mechanisms crucial for providing genetic counseling, reproductive decision-making, and developing targeted therapies and prognostic predictions. KFS exhibits high genetic heterogeneity, and its etiology remains incompletely understood, generally attributed to a multifactorial disease involving predominant genetic factors and environmental influences [9], possibly following an oligogenic inheritance pattern [10]. The widespread adoption of high-throughput sequencing technologies, coupled with the accumulation and exploration of clinical genetic resources, provides a pathway for investigating the genetic basis of sandwich fusion. Therefore, we conducted a WES analysis of 68 AAD patients with a genetic background of sandwich fusion, identifying rare genomic variations, and performed comparative analyses using exome data from a threefold number of healthy individuals.

## 2. Methods

### 2.1. Human Subject Recruitment and Demographic Data Collection

Between January 2016 and January 2022, patients with AAD undergoing orthopedic surgery at Peking University Third Hospital were invited to participate in genetic research if they had a genetic background predisposing them to sandwich fusion. A total of 68 patients with sandwich fusion were recruited, and clinical diagnoses were verified by two senior orthopedic physicians and one radiologist. Demographic information, detailed family medical history, physical examination results, clinical symptoms, and surgical details were collected. Radiological assessments included anteroposterior and lateral radiographs, preoperative and postoperative MRI evaluations of AAD, spinal cord compression, congenital fused vertebral segments, Samartzis classification of KFS, spinal deformities, and other associated anomalies. A total of 219 WES datasets of unrelated Chinese individuals without musculoskeletal or spinal disorders were selected as controls from Berry's in-house database, with similar sex ratios and age distributions.

### 2.2. Whole-Exome Sequencing and Data Analysis

Informed consent was obtained from all patients and guardians of minors, and approval was obtained from the Peking University Third Hospital Medical Ethics Committee. Peripheral blood samples were collected from 68 unrelated patients with sandwich fusion during general anesthesia, and sequencing was performed on the Novaseq6000 platform (Illumina, San Diego, USA) in 150 bp paired-end reads mode by Berry Genomics Incorporated, China. After quality assessment, sequencing reads were aligned to the Human Genome 38 (hg38/GRCh38) by BWA software, and variants calling was by GATK software. Based on the American College of Medical Genetics and Genomics (ACMG) guidelines [11], further annotation and interpretation were conducted by ANNOVAR (http://annovar.openbioinformatics.org/en/latest/) and the Enliven Variants Annotation Interpretation System authorized by Berry Genomics, annotation databases mainly included gnomeAD (http://gnomad.broadinstitute.org/), the 1000 Genomes Project (http://browser.1000genomes.org), HGMD (http://www.hgmd.org), and HPO (https://hpo.jax.org/app/). Variant deleteriousness was provided by prediction algorithms in silico such as SIFT (http://sift.jcvi.org), MutationTaster (http://www.mutationtaster.org/), and CADD (http://cadd.gs.washington.edu). Minor allele frequency (MAF) < 0.001 was the criterion of a rare variant, and Combined Annotation Dependent Depletion (CADD) predicted score of 15 or more was the criterion of a skeptically deleterious variant. Variant was considered to be diagnostic when its reported phenotypes aligned with clinical findings in this study as well as a consistent inheritance mode.

### 2.3. Statistical Analysis of Mutational Burden

A control group consisting of irrelevant 219 individuals without skeletal or spinal disorders underwent exome sequencing. Within the exomes, we compared the burden of all deleterious rare mutations (CADD score > 15) at the gene level between the patients group and the control group. The ranking of potential pathogenic genes was based on the odds ratio (OR values). Statistical analysis was conducted using SPSS 26.0 software, with Fisher's exact test for comparing rates between the two groups, considering *P* < 0.05 as statistically significant as well as recalculating adjusted *P* values to make the results more credible.

## 3. Results

### 3.1. Clinical Characteristics and Demographic Information

The study included 68 unrelated individuals with sandwich fusion and 219 unrelated individuals without musculoskeletal disorders, and their detailed demographic information is presented in Table 1. In the case group, the average age at diagnosis was 44 years, with the majority of cases occurring between 36 and 60 years old. All 68 patients exhibited craniovertebral junction anomalies (C0-C1 fusion) and C2-C3 fusion, with C6-C7 being the most common fusion level besides these. Samartzis et al. [12] classified KFS into three types based on the continuity of the fused segments: type I (single fusion), type II (two or more noncontiguous fusions), and type III (two or more contiguous fusions). In this study, type II accounted for 85%, and type III accounted for 15%. All patients presented with AAD and basilar invagination. Other spinal and spinal cord anomalies included 18 cases of Chiari malformation, 16 cases of scoliosis, 11 cases of syringomyelia, 9 cases of torticollis, and 4 cases of os odontoideum. Additionally, 11.8% of patients with sandwich fusion exhibited cardiac or vascular morphological abnormalities, indicating a close association with congenital cardiovascular malformations [13].

### 3.2. Variants Searched in Reported KFS Causal Gene

There was no diagnostic variant found, and then we searched all genuine variants in 68 people for the presence of four reported KFS pathogenic genes [14–17] to search for potentially explicable pathogenic variants. The search results showed that, as shown in Table 2, the occurrence rate of these gene loci in sandwich fusion patients varied greatly, with the highest mutation frequency being *MYO18B* and the smallest mutation frequency being *GDF6*.

### 3.3. Genetic Burden Analysis between Cases and Controls

Methods such as next-generation sequencing and burden analysis based on high-throughput sequencing have been used to parse the underlying genetic mechanisms of rare skeletal diseases [18]. By comparing all rare variants with high pathogenic potential (MAF < 0.001 and CADD predictive value > 15) between the case group (*n* = 68) and the control group (*n* = 219), we calculated that, at the exon level, compared with the healthy control group, the mutant genes significantly enriched in the case group were *KMT5A* (odds ratio = 112.89673), *HYDIN* (odds ratio = 29.02934), and *PCDHB4* (odds ratio = 17.09137) as shown in Table 3.

### 3.4. Individuals with Multiple Mutations and Potential Oligogenic Effects

**Figure 1:**
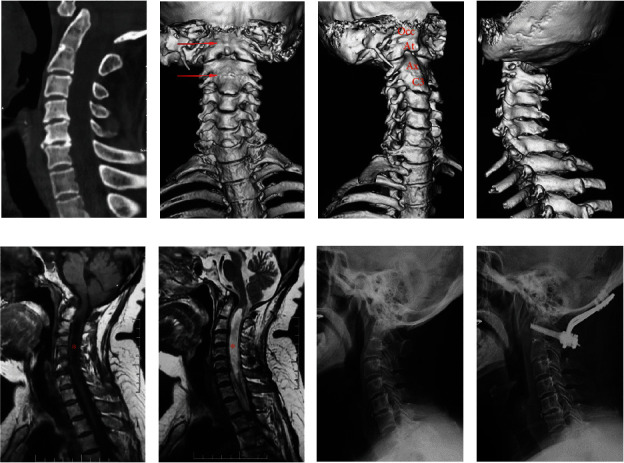
One 62-year-old woman (patient ID 22KAH0010) with quadriplegia carried multiple nonsynonymous mutations in *MEOX1* and *KMT5A*. (a–d) the radiological imaging showed sandwich fusion that is occiput-atlas fusion combined with axis-C3 vertebra fusion. The areas pointed out by the red arrows in b are the fusion sites. (e, f) MRI showed AAD compressing the spinal cord as well as a huge syringomyelia where the red pentastars indicate. (g) Preoperative lateral X-ray films showed sandwich fusion, AAD, and swan neck deformity. (h) Postoperative lateral X-ray films showed atlantoaxial joint was in anatomical reduction.

We listed seven patients with severe clinical manifestations and analyzed their genetic mutations in detail. In these patients, we found complex mutations with multiple viable genes, including *KMT5A*, *HYDIN*, *PODXL*, *MEOX1*, and *MYO18B*, which may be the oligogenic effect of KFS [10]. Remarkably, they all carry a mutation in *MEOX1*. The splicing mutation c.1-19G>A was found in two patients, the splicing mutation c.420+133_420+151del was found in three patients, and the splicing mutation c.420+146T>A was observed in two patients. Some typical individuals who carry multiple mutations in these genes are listed in Table 4 separately with the CADD prediction score, and one of them gives her consent to demonstrate her imaging details in Figure 1.

## 4. Discussion

Klippel-Feil syndrome patients exhibit diverse clinical manifestations, and the most common fusion site is C2-C3 level (74.1%); sandwich fusion occurs if accompanied by atlanto-occipital ossification. It contributes to a higher risk of AAD and severe cervical spinal cord disease, emphasizing the importance of recognizing this specific subtype. Due to the loss of mobility at the fusion level, the compensatory stress is concentrated in the atlantoaxial joint and increases the stretch of the atlantoaxial cruciate ligament, thus accelerating its degeneration and increasing the risk of AAD in the long term [19]. Previous research comparing sandwich AAD patients with nonsandwich AAD patients revealed an earlier onset of disease, more severe neurological impairments, and a higher prevalence of other deformities. Additionally, anomalies in the vertebral artery and internal carotid artery, along with craniovertebral junction deformities, are prevalent in sandwich-type AAD patients, posing challenges for surgical treatment and resulting in poorer outcomes [20]. In this study, 68 cases of sandwich fusion patients queue statistics show that merge proportion (63.2%), scoliosis, spinal cord disease (23.6%), syringomyelia, and a high proportion (16.1%) once again confirmed that the sandwich fusion is a subtype of KFS with well-marked clinical features.

The genetic heterogeneity of KFS is another distinctive feature [9]. Currently, four pathogenic genes with inheritance patterns, *GDF3* and *GDF6* (autosomal dominant) and *MEOX1* and *MYO18B* (autosomal recessive), have been identified in families with congenital cervical fusion. However, these genes only explain less than 10% of KFS cases. In this study, the seven individuals with severe sandwich fusion all carried nonsynonymous mutations in *MEOX1* and *KMT5A*, suggesting a close association between these genes and the occurrence of sandwich fusion. *MEOX1*, a bHLH-type transcription factor involved in vertebral development, has been linked to vertebral defects in zebrafish and mice, exhibiting phenotypes similar to human KFS [21]. In this study, *MEOX1* gene mutations were correlated with the severity of *AAD*, younger age of onset, and severity of cervical spinal cord disease, indicating a potential involvement of *MEOX1* mutations in the pathogenesis of sandwich fusion.

Lysine methyltransferase 5A (*KMT5A*), also known as *SET8*, is located on the long arm of chromosome 12 (12q24.31). *KMT5A* catalyzes specific monomethylation of lysine 20 on histone H4, participating in various cellular processes such as gene transcription regulation, replication origin regulation, genome stability maintenance, and cell cycle regulation. Recent studies have linked *KMT5A* to various cancers, indicating its role in promoting gene expression and cell proliferation through signaling pathways like WNT and p53 [22]. Therefore, we reasonably speculate that dysfunction of *KMT5A* during embryonic development may lead to disturbances in craniovertebral junction development and abnormal spinal segmentation. The instability of *KMT5A* in these individuals may also make them more susceptible to tumorous diseases, warranting attention to preventive measures and monitoring.

Genetic diseases affecting the musculoskeletal system present high heterogeneity both clinically and genetically, complicating diagnosis and treatment. Genome-wide association studies (GWAS) have identified 463 genes associated with human skeletal disorders [23], but there is still limited research on many rare subtypes. Our study established the first genetic sequencing cohort for the sandwich fusion subtype of KFS, representing a sizable genetic cohort for KFS [10]. The findings provide crucial insights into the etiology, diagnostics, and therapeutics of this condition.

The main limitation of this study is the relatively small sample size of the discovery set, which is due to the rarity of sandwich fusion. Besides, single genes cannot fully explain the occurrence and pathology of this disease considering its genetic heterogeneity and complex associations with the pregnancy environment [24], and we have not been considered environmental factors in this study. Furthermore, all the genetic findings are not validated in the verification set or in experimental animal.

## 5. Conclusions

In summary, we constructed the first genetic sequencing cohort for the sandwich fusion anomaly and analyzed its genetic basis at the exome level, identifying several new disease-related genes and proposing a potential hypothesis. Though the findings contribute valuable information for the etiology of KFS, the rarity of sandwich fusion poses challenges in sample collection, and further research is required to comprehensively understand genetic and environmental factors.

## Figures and Tables

**Table 1:** Demographic features of patients with sandwich fusion.

Clinical information	Case number	Proportion	Control number	Proportion
Gender				
Female	43	63%	138	63%
Male	25	37%	81	37%
Age level				
0-18	6	8.8%	0	0
18-35	11	16.2%	43	19.6%
36-60	39	57.4%	133	60.7%
>60	12	17.6%	43	19.6%
Fusion level				
C0-1	68	100%	—	—
C1-2	0	0	—	—
C2-3	68	100%	—	—
C3-4	4	5.9%	—	—
C4-5	5	7.4%	—	—
C5-6	4	5.9%	—	—
C6-7	9	13.2%	—	—
C7-t1	2	2.9%	—	—
T1-12	1	1.4%	—	—
T12-L5	1	1.4%	—	—
Samartzis classification				
KFS I	0	0	0	0
KFS II	58	85%	0	0
KFS III	10	15%	0	0
Combined malformations				
Atlantoaxial dislocation	68	100%	0	0
Basilar invagination	68	100%	0	0
Chiari malformation	18	26.5%	0	0
Cervical spondylotic myelopathy	43	63.2%	0	0
Cardiac anomalies	8	11.8%	0	0
Os odontoideum	4	5.9%	0	0
Syringomyelia	11	16.1%	0	0
Scoliosis	16	23.5%	0	0
Torticollis	9	13.2%	0	0
Fixed level in surgery				
C0-2	34	50%	—	—
C0-3	28	41.2%	—	—
C0-4	3	4.4%	—	—
C0-5	1	1.5%	—	—
Total	68	100%	219	100%

**Table 2:** Searching results among reported KFS-related genes in 68 patients.

KFS causal genes	Patients included	Carrier rate	SNP counts
*GDF6*	3	4.4%	3
*MEOX1*	9	13.2%	9
*GDF3*	42	61.8%	84
*MYO18B*	42	61.8%	1438

**Table 3:** Burden analysis and the top 3 genes ranked by odds ratio.

Genes	Cases	Cases included	Cases excluded	Controls	Controls included	Controls excluded	Odds ratio	*P* value	Adjusted *P* value
*KMT5A*	68	42	26	219	3	216	112.90	0.000000000000000000000000000087	8.65*E* − 29
*HYDIN*	68	42	26	219	4	215	84.24	0.00000000000000000000000000089	8.91*E* − 28
*PODXL*	68	12	56	219	2	217	22.92	0.000000846	8.45*E* − 07

**Table 4:** Individuals with multiple mutations and their severe clinical manifestations.

Case ID	Symptoms	Genes	dbSNP	Variant type	Variants	MAF	CADD
22KAH0010	AAD, AOZ, BI, KFS-SW, CSM, syringomyelia, and cardiac anomalies	*MEOX1*	rs536283503	UTR3	c.420+133_420+151del	0	—
		*KMT5A*	rs61955127	Missense	c.995T>C:p.l332p	0	31
22KAH0018	AAD, AOZ, BI, CM, KFS-SW, and CSM	*MEOX1*	rs536283503	UTR3	c.420+133_420+151del	0	—
		*KMT5A*	rs61955127	Missense	c.995T>C:p.l332p	0	31
22KAH0006	AAD, AOZ, BI, KFS-SW, CSM, and SCD	*MEOX1*	rs536283503	UTR3	c.420+133_420+151del	0	—
		*KMT5A*	rs61955127	Missense	c.995T>C:p.l332p	0	31
22KAE00005	AAD, AOZ, BI, KFS-SW, CSM, cardiac anomalies, and nuchal ligament ossification	*MEOX1*	rs142430146	UTR5	c.1-19G>A	0.00131754	—
22KAE00057	AAD, AOZ, BI, KFS-SW, CSM, and nerve root sheath cyst	*MEOX1*	rs142430146	UTR5	c.1-19G>A	0.00131754	—
22KAH0009	AAD, AOZ, BI, KFS-SW, CSM, and ICAA	*MEOX1*	rs1033265959	UTR3	c.420+146T>A	0	—
22KAH0005	AAD, AOZ, BI, CM, KFS-SW, and CSM	*MEOX1*	rs1033265959	UTR3	c.420+146T>A	0	—
		*KMT5A*	rs61955127	Missense	c.995T>C:p.l332p	0	31

Abbreviations: AAD: atlantoaxial dislocation; AOZ: atlas occipitalization; BI: basilar invagination; CM: Chiari malformation; CSM: cervical spinal myelopathy; ICAA: internal carotid artery aneurysm; KFS-SW: sandwich fusion deformity subtype in Klippel-Feil syndrome; SCD: spondylocostal dysostosis.

## Data Availability

Datasets are available from the corresponding author or browsed on the GSA website (https://ngdc.cncb.ac.cn/gsa/browse) by no. HRA005944.
